# Clonal Hematopoiesis (CHIP) in Pulmonary Embolism and CTEPH: Evidence, Mechanisms, and Risk Stratification

**DOI:** 10.3390/ijms27062750

**Published:** 2026-03-18

**Authors:** Lukasz Szarpak, Monika E. Jach, Michal Skoczylas, Sebastian Radej, Michal Pruc

**Affiliations:** 1 Institute of Medical Science, Collegium Medicum, The John Paul II Catholic University of Lublin, 20-708 Lublin, Poland; michal.pruc@kul.pl; 2 Henry JN Taub Department of Emergency Medicine, Baylor College of Medicine, Houston, TX 77030, USA; 3 Institute of Biological Sciences, Collegium Medicum, The John Paul II Catholic University of Lublin, 20-708 Lublin, Poland; monika.jach@kul.pl (M.E.J.); michal.skoczylas@kul.pl (M.S.); sebastian.radej@kul.pl (S.R.)

**Keywords:** CHIP, CTEPD, CTEPH, fibrinolysis, immunothrombosis, inflammasome, neutrophil extracellular traps (NETs), pulmonary embolism, thrombus resolution, variant allele frequency (VAF)

## Abstract

Pulmonary embolism (PE) is biologically heterogeneous. Despite guideline-directed anticoagulation, a subset of patients develops recurrent venous thromboembolism, persistent exertional limitation, residual perfusion defects, and progression to chronic thromboembolic pulmonary disease (CTEPD) or chronic thromboembolic pulmonary hypertension (CTEPH). Conventional risk factors explain much of the index event but incompletely account for thrombus non-resolution and chronic sequelae. Clonal hematopoiesis of indeterminate potential (CHIP)—the age-associated expansion of hematopoietic clones carrying somatic mutations—defines a measurable thrombo-inflammatory endophenotype that is strongly genotype- and clone-size (variant allele frequency; VAF)-dependent. Across human studies, JAK2-CHIP and TET2-CHIP show the most consistent associations with VTE/PE, whereas isolated DNMT3A-CHIP is frequently neutral, and larger clones tend to confer stronger effects. Mechanistically, CHIP can bias myeloid cells toward inflammasome/IL-1β signaling and endothelial activation, increase monocyte tissue factor activity, and promote immunothrombosis with neutrophil extracellular trap (NET) formation. NET-rich thrombi may adopt a dense fibrin–DNA–histone architecture that resists endogenous fibrinolysis, favoring organization and persistence. CTEPH offers a translational window to interrogate this model because thrombotic material and deep phenotyping are accessible. We synthesize genotype- and VAF-resolved clinical and mechanistic evidence using a structured strength-of-evidence framework and propose a pragmatic phenotyping roadmap with testable predictions for prospective post-PE validation. CHIP testing in PE/CTEPH remains investigational and should not currently change standard care.

## 1. Introduction

From a clinical standpoint, pulmonary embolism (PE) is stratified according to early mortality risk and anticipated response to therapy [1]. Despite guideline-based anticoagulation, a subset of patients develops recurrent VTE, persistent functional limitation, chronic perfusion defects, or progresses to chronic thromboembolic pulmonary disease (CTEPD) and chronic thromboembolic pulmonary hypertension (CTEPH)—potentially curable entities when recognized in time [2,3,4].

These outcomes expose a gap in our models: beyond determinants of the index event, post-PE care requires understanding on why some thrombi fail to resolve. Thrombus non-resolution is shaped by clot microstructure and by the local vascular-immune milieu (fibrin architecture, DNA/histone content, platelet–leukocyte crosstalk, and the balance between endogenous fibrinolysis and organization/fibrosis). Clonal hematopoiesis of indeterminate potential (CHIP) is an age-associated, clinically detectable state that can reprogram myeloid cells toward thrombo-inflammation (inflammasome signaling, tissue factor expression, and neutrophil extracellular trap (NET) formation).

**Figure 1:**
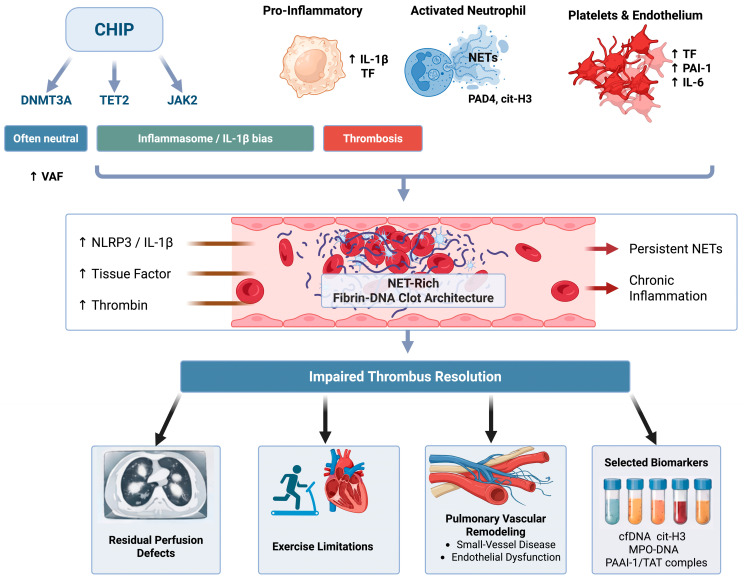
Hypothesized pathway from CHIP to non-resolving post-PE thrombus. Pro-inflammatory myeloid priming, NETosis, and platelet/endothelial activation converge on NET-rich fibrin–DNA thrombi, sustaining inflammation and reducing fibrinolytic clearance. Clinical consequences include residual perfusion defects, functional limitation, pulmonary vascular remodeling, and measurable biomarkers (cfDNA, cit-H3, MPO–DNA, PAI-1, TAT complexes). Abbreviations: CHIP, clonal hematopoiesis of indeterminate potential; VAF, variant allele frequency; PE, pulmonary embolism; NETs, neutrophil extracellular traps; TF, tissue factor; PAI-1, plasminogen activator inhibitor-1; cfDNA, cell-free DNA; cit-H3, citrullinated histone H3; MPO, myeloperoxidase; TAT, thrombin–antithrombin.

We use the PE-to-CTEPD/CTEPH continuum as a human model of thrombus non-resolution and interpret the literature through a genotype- and clone-size-resolved framework (variant allele frequency, VAF). Central hypothesis (hypothesis-generating): higher-burden, high-impact genotypes (most consistently TET2 and JAK2, in a VAF-dependent manner) primarily act as modifiers of thrombus composition and resolution biology after PE, predisposing to fibrinolysis resistance, organization, and chronic obstruction. We do not claim causality; instead, we propose a falsifiable model intended to generate prospective predictions for post-PE cohorts. We summarize the working model linking CHIP (genotype and VAF) to thrombo-inflammation, NET-rich fibrin–DNA architecture, and impaired thrombus resolution leading to CTEPD/CTEPH phenotypes in Figure 1.

What this review adds (why this topic matters now):

- Applies a genotype- and clone-size (VAF) framework to the PE-to-CTEPD/CTEPH continuum (thrombus non-resolution), rather than to incident VTE alone.
- Integrates clinical evidence with molecular mechanisms linking CHIP-driven myeloid bias to immunothrombosis, NET-rich fibrin architecture, and impaired fibrinolysis.
- Provides testable, mechanism-resolved predictions and a pragmatic roadmap for prospective cohorts (minimal endpoints, confounders, and candidate targets) to validate CHIP-informed risk stratification and therapy concepts.

Throughout, we explicitly distinguish evidence for incident VTE/PE in large cohorts (Level 1) from the more limited, PE/CTEPD/CTEPH-specific evidence (Level 2) and from mechanistic/experimental data that provide biological plausibility (Level 3), to separate what is established from what remains hypothesis-driven.

## 2. Methods

### 2.1. Review Design and Scope

This manuscript is a structured narrative review synthesizing clinical and translational evidence linking clonal hematopoiesis of indeterminate potential (CHIP) with PE and post-PE chronic thromboembolic disease (CTEPD/CTEPH). Our aim was scoping and triangulation across population cohorts, PE/CTEPD/CTEPH deep-phenotyping studies, and mechanistic data, with claims organized in an evidence-strength map at three nested levels, rather than exhaustive capture of every report. We aimed to (i) distinguish determinants of incident VTE/PE from mechanisms plausibly contributing to thrombus persistence and chronicity, (ii) integrate genotype- and clone-size (variant allele frequency; VAF)-resolved human evidence with mechanistic data, and (iii) derive testable, genotype-informed research predictions. To improve transparency and reproducibility, we report information sources, search logic, study selection principles, and the approach to evidence synthesis. Given heterogeneity in study designs, sequencing platforms, endpoints, and analytic strategies, we did not perform a quantitative meta-analysis.

### 2.2. Information Sources and Search Dates

We searched PubMed/MEDLINE as the primary database and used Embase and Web of Science as complementary sources to broaden coverage across hematology, cardiovascular medicine, thrombosis, and translational biology. Searches were performed from database inception to the most recent update prior to manuscript finalization (last search: January 2026). To minimize the risk of missing relevant evidence, we also screened the reference lists of key primary studies and relevant reviews (backward citation searching) and considered additional eligible records identified through this process.

### 2.3. Search Strategy

The search strategy was structured around three concept blocks: (1) clonal hematopoiesis/CHIP and somatic mutations (including recurrent CHIP driver genes); (2) venous thromboembolism phenotypes (VTE, pulmonary embolism/PE, and recurrence); and (3) chronic thromboembolic sequelae and thrombus biology (including thrombus persistence/organization and immunothrombosis-related pathways). Terms were combined using Boolean operators and adapted to each database using controlled vocabulary where applicable (e.g., MeSH in PubMed and Emtree in Embase), supplemented by free-text keywords. A representative search formulation included the following: (“clonal hematopoiesis” OR “CHIP” OR “ARCH” OR “somatic mutation*” OR DNMT3A OR TET2 OR JAK2 OR ASXL1) AND (“venous thromboembolism” OR “VTE” OR “pulmonary embolism” OR “PE” OR recurrence) AND (“CTEPH” OR “CTEPD” OR “chronic thromboembolic” OR “post-PE” OR “thrombus resolution” OR “thrombus persistence” OR “thrombus organization” OR “immunothrombosis” OR “neutrophil extracellular trap*” OR “NET*” OR fibrinolysis). We intentionally favored sensitivity to capture both clinical association studies and mechanistic/translational work relevant to thrombus organization and persistence; study selection and evidence grading are described below. The complete, reproducible database-specific search strings for all sources are provided in Supplementary Table S1.

### 2.4. Eligibility Criteria

We included the following: (i) human studies in adults reporting CHIP prevalence and/or associations between CHIP and venous thromboembolism phenotypes, including incident VTE/PE, recurrent events, and PE-related chronicity endpoints (e.g., post-PE persistence, residual obstruction/perfusion defects, CTEPD/CTEPH), and (ii) clinically relevant experimental or translational studies that directly informed thromboinflammatory mechanisms plausibly linking CHIP to thrombosis and thrombus persistence (e.g., ex vivo thrombus/tissue analyses, multi-omics, functional studies in primary cells, and selected animal models interrogating CHIP-relevant pathways such as immunothrombosis, thrombus organization, and fibrinolysis resistance). For clinical association studies, we prioritized reports with clearly defined CHIP ascertainment (sequencing approach and/or reporting threshold) and adjudicated or clearly specified VTE/PE outcomes.

We excluded single-patient case reports unless they provided unique tissue-level or mechanistic data that materially informed the conceptual model. Conference abstracts without full text were not used as primary evidence; when cited to illustrate emerging observations where full data were unavailable, they were explicitly labeled as preliminary and not used to support higher-certainty conclusions. Studies in pediatric populations, non-human studies without clear translational relevance to CHIP-associated pathways, and reports lacking sufficient methodological detail to interpret CHIP status or outcomes were not considered for evidence synthesis.

### 2.5. Study Selection

All retrieved records were imported into a reference manager and deduplicated prior to screening. Titles and abstracts were screened for relevance to CHIP/clonal hematopoiesis and thromboembolic outcomes (VTE/PE, recurrence) or thrombus biology pertinent to PE-related chronicity (including CTEPD/CTEPH and thrombus persistence/organization). Full texts were obtained and assessed when eligibility was plausible based on the abstract. We screened n = 233 records at the title/abstract level and assessed n = 97 full texts, resulting in n = 50 included studies.

When multiple publications reported overlapping cohorts, we prioritized the most comprehensive report (largest sample size and/or longest follow-up and clearest endpoint definitions) and used additional publications from the same cohort only for complementary analyses (e.g., deeper phenotyping, genotype/VAF stratification, or mechanistic/translational endpoints). The selection process was designed to be transparent and reproducible; however, given the narrative/translational scope of the review, it is not presented as a formal systematic review workflow.

### 2.6. Data Charting and Extracted Items

For included primary human studies, we charted: study design; setting and population; sample size; outcome definitions and follow-up; sequencing approach (targeted panel vs. exome/genome, depth, and VAF threshold); CHIP genotype distribution (including co-mutations and multiplicity); clone-size metrics (VAF categories or continuous analyses); adjustment variables and potential confounders (age, sex, cancer/therapy exposure, cardiovascular disease, inflammatory comorbidities, anticoagulation and other relevant treatments); and effect estimates where reported. For translational and experimental studies, we charted model system; key molecular pathways (e.g., inflammasome/IL-1β/IL-6, tissue factor, NETosis, endothelial activation); thrombus composition/architecture features; and measures linked to organization, persistence, or fibrinolysis resistance.

### 2.7. Evidence Integration and Strength-of-Evidence Framework

**Figure 2:**
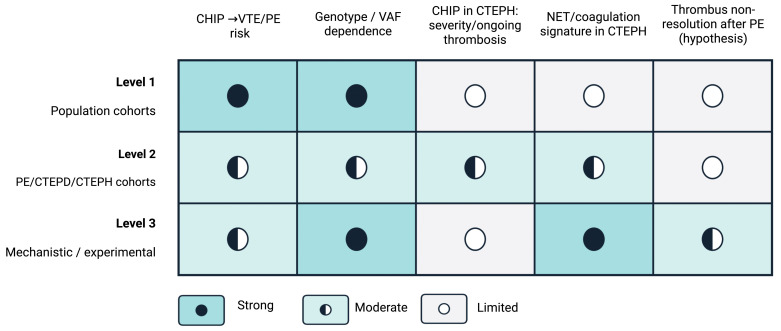
Strength of evidence linking clonal haematopoiesis (CHIP) to venous thromboembolism and the post-PE/CTEPH continuum across study levels. Abbreviations: CHIP, clonal haematopoiesis of indeterminate potential; VTE, venous thromboembolism; PE, pulmonary embolism; CTEPD, chronic thromboembolic pulmonary disease; CTEPH, chronic thromboembolic pulmonary hypertension; VAF, variant allele frequency; NET, neutrophil extracellular trap(s).

We synthesized evidence using a structured narrative approach emphasizing genotype- and VAF-resolved signals and endpoints extending beyond incident VTE/PE, particularly recurrence and post-PE chronicity (residual perfusion defects, CTEPD/CTEPH) when available. The following framework and summary tables/figures are author-synthesized tools to structure and communicate the evidence; they are not intended as guideline-level recommendations or prescriptive clinical decision rules. They are provided to facilitate interpretation, highlight consistency/uncertainty, and identify testable hypotheses. To avoid over-interpretation of associative data and to maintain clear separation between clinical evidence and mechanistic plausibility, we organized conclusions using three nested evidence levels: Level 1—robust population associations (large cohorts with adjusted analyses); Level 2—PE/CTEPD/CTEPH-relevant clinical cohorts with deep phenotyping (imaging, hemodynamics, recurrence or post-PE persistence endpoints); and Level 3—mechanistic/translational evidence (cell/animal models, thrombus/tissue biology, transcriptomics/proteomics) supporting biological plausibility and generating testable predictions. Statements that rely primarily on associative evidence were phrased conservatively, and causal language was avoided unless supported by convergent evidence across levels. To contextualize causality versus association, we apply a three-level evidence framework (population, disease-specific cohorts, and mechanistic/experimental studies), summarized in Figure 2.

### 2.8. Risk of Bias Considerations

Because included studies varied substantially in sampling frames, sequencing sensitivity, CHIP definitions (including VAF thresholds), confounder adjustment, and outcome ascertainment, we did not apply a single numeric risk-of-bias score. Instead, we qualitatively weighted evidence toward studies with clearly defined outcomes; robust adjustment for age and cancer/therapy exposure; genotype- and VAF-stratified analyses; and clinically meaningful endpoints. Limitations arising from residual confounding, selection bias (e.g., referral CTEPH cohorts), and measurement heterogeneity were explicitly noted in the synthesis.

### 2.9. Reporting and Deviations

This review is intended as a structured narrative synthesis rather than a formal systematic review and therefore does not include a PRISMA flow diagram or pooled effect estimates. Any deviations from the above approach (e.g., inclusion of unique mechanistic case material) are explicitly signposted in the relevant sections.

## 3. CHIP: Definition, Epidemiology, and Why Genotype Matters

CHIP is defined as the presence of somatic mutations in genes recurrently implicated in myeloid neoplasms, detected in blood cells of individuals without an overt hematologic malignancy, with a variant allele frequency (VAF) of at least 2% [5,6,7]. The 2% threshold is an operational criterion; modern sequencing platforms can detect clones well below this level, but the clinical significance of very small clones remains less certain [7,8,9].

CHIP should be distinguished from clonal cytopenia of undetermined significance (CCUS). CHIP denotes clonal mutations in the absence of unexplained cytopenias and dysplasia, whereas CCUS combines a clonal mutation with persistent unexplained cytopenias [10]. This distinction is prognostically important: CCUS carries a substantially higher risk of progression to myeloid malignancy than CHIP, and typically warrants closer hematologic follow-up [11].

CHIP is strongly age-associated, reported in more than 10% of individuals older than 70 years and in 20–30% or more of those in their eighth and ninth decades, depending on sequencing depth, gene panels, and VAF thresholds [5,6,11,12,13,14]. This is directly relevant to PE and CTEPH, which disproportionately affect older patients, raising the possibility that clonal hematopoiesis may be a frequent background modifier of thrombo-inflammatory risk [15,16,17].

The most commonly involved genes in population cohorts are DNMT3A (approximately 40–50% of CHIP), TET2 (approximately 20–30%), and ASXL1 (approximately 18–20%), with less frequent involvement of JAK2, PPM1D, TP53, and spliceosome genes (SF3B1, SRSF2, U2AF1) [5,8,11,13,18]. Importantly, risk is not evenly distributed across genotypes: JAK2 and TET2 tend to show the clearest association with thrombotic events, whereas isolated DNMT3A often confers a weaker or neutral signal for PE/VTE in population data [14,19,20,21].

VAF provides an estimate of clone size and, by extension, biological “dose”. Larger clones (commonly VAF ≥ 10%) appear to carry greater systemic impact, particularly across inflammatory and pro-thrombotic pathways [19,22,23]. Table 1 provides a pragmatic guide to interpreting CHIP results in PE/CTEPD/CTEPH by integrating genotype, clone size (VAF), and clinical context, and highlights common interpretation pitfalls. Longitudinal dynamics may matter more than a single measurement; increasing VAF suggests clonal expansion and potentially rising risk [22,23].

Mechanistically, several CHIP genotypes converge on innate immune activation. TET2 and DNMT3A mutations are linked to NLRP3 inflammasome activation and increased IL-1β/IL-6 signaling in macrophages, promoting a pro-inflammatory, pro-thrombotic milieu [5,8,15,18,24]. JAK2 (particularly JAK2V617F) is associated with marked thrombotic propensity, integrin activation, and enhanced NET formation [8,14,15,24].

**Table 1:** Interpreting CHIP in PE/CTEPD/CTEPH: genotype, VAF, and clinical context.

Result Element	Typical VTE/PE Signal	Dominant Biology/Mechanism	Potential Relevance in PE/CTEPD/CTEPH	Key Interpretation Pitfall
A. Genotype (driver mutations)				
JAK2 (JAK2-CHIP)	High	MPN-like prothrombotic myeloid–platelet biology; increased tissue factor (TF) and NET propensity.	Recurrent or disproportionate VTE/PE; atypical thrombotic phenotypes; may inform hematology co-management and trial enrichment.	Exclude occult MPN; consider hematology referral (especially with abnormal counts or splenomegaly).
TET2 (TET2-CHIP)	Moderate (often reproducible)	Clonal inflammation (inflammasome/IL-1β bias); cytokine upregulation; immunothrombosis/NETs.	Older/unprovoked PE; hypothesis-generating link to thrombus non-resolution and chronic sequelae across the CTEPD/CTEPH spectrum.	Association ≠ causality; limited prospective PE validation—avoid clinical decisions outside research.
DNMT3A (DNMT3A-CHIP)	Low/uncertain for PE	Heterogeneous; often an aging marker with mild inflammatory tone (context- and VAF-dependent).	More likely a background modifier than a primary driver of PE/CTEPH; may matter in combination with other risks.	Do not assign ‘high risk’ based on DNMT3A alone; effects may reflect age/comorbidity confounding.
ASXL1 (ASXL1-CHIP)	Variable	Context-dependent (often linked to exposures such as smoking); inflammatory bias varies by comorbidity burden.	Interpret within environmental/clinical risk context; may act as a modifier in high-risk groups.	High confounding risk (smoking/comorbidities); requires careful adjustment in cohort analyses.
B. Clone size and dynamics (variant allele frequency, VAF)				
Low VAF (<5%)	Limited/gene-dependent	Small clone; uncertain biological ‘dose’.	May represent background clonal hematopoiesis; relevance depends on genotype and context.	Avoid overinterpretation; confirm assay thresholds and consider repeat testing if needed.
Medium VAF (5–10%)	Potentially meaningful	Intermediate clone burden; may cross a threshold for measurable thrombo-inflammatory effects (genotype-dependent).	May help stratify risk and enrich cohorts/trials (consistent with Figure 3) when paired with phenotype/biomarkers.	Still context-dependent (gene, comorbidities, anticoagulation); assay standardization matters.
High VAF (>10%) and/or rising on serial testing	More likely clinically relevant	Higher clone burden; supports dose–response biology; rising VAF suggests clonal expansion.	Higher likelihood of thrombo-inflammatory endophenotype; may prioritize deep phenotyping (Figure 4) and trial enrichment.	Use standardized assays and repeat measurements; assess co-mutations/multiple drivers and hematologic red flags.
C. Hematologic red flags and clinical context				
Cytopenias or atypical blood counts/morphology	Not applicable	May indicate CCUS/MDS/MPN rather than ‘isolated’ CHIP.	Different risk profile and management; may shift interpretation of thrombotic risk and prognosis.	Not ‘pure CHIP’—needs hematology work-up; do not extrapolate CHIP-only data.
Clinical context (age, chronic inflammation, atherosclerosis, cancer, smoking)	Modifier/confounder	Can amplify thrombo-inflammation or confound CHIP-outcome associations.	May contribute to thrombus persistence/non-resolution; key covariates in PE/CTEPH phenotyping (Figure 4).	High confounding risk; requires multivariable models and harmonized phenotyping in prospective studies.

Abbreviations: ASXL1, additional sex combs like 1; CCUS, clonal cytopenia of undetermined significance; CHIP, clonal haematopoiesis of indeterminate potential; CTEPD, chronic thromboembolic pulmonary disease; CTEPH, chronic thromboembolic pulmonary hypertension; DNMT3A, DNA methyltransferase 3A; IL-1β, interleukin-1 beta; JAK2, Janus kinase 2; MDS, myelodysplastic syndrome(s); MPN, myeloproliferative neoplasm(s); NET, neutrophil extracellular trap(s); PE, pulmonary embolism; TF, tissue factor; TET2, tet methylcytosine dioxygenase 2; VAF, variant allele frequency; VTE, venous thromboembolism.

**Figure 3:**
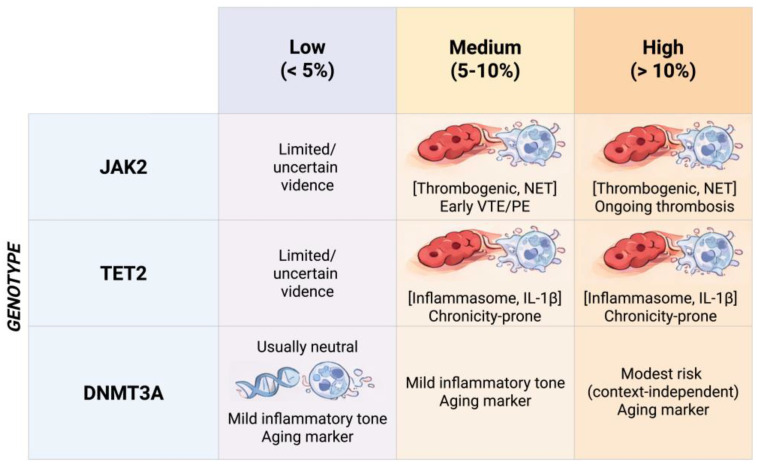
Genotype–clone size framework for interpreting CHIP in the thromboembolic continuum. Abbreviations: CHIP, clonal haematopoiesis of indeterminate potential; IL-1β, interleukin-1 beta; JAK2, Janus kinase 2; DNMT3A, DNA methyltransferase 3A; NET, neutrophil extracellular trap(s); PE, pulmonary embolism; VTE, venous thromboembolism.

**Figure 4:**
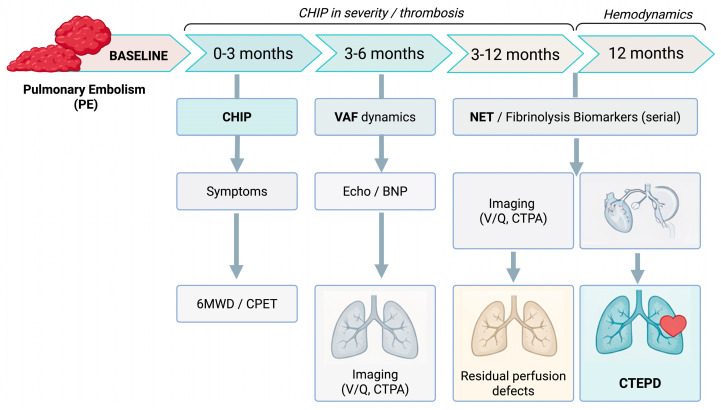
Proposed post-PE follow-up framework integrating clonal haematopoiesis (CHIP) assessment with serial biomarkers and imaging to identify patients at risk for CTEPD/CTEPH. Abbreviations: 6MWD, six-minute walk distance; BNP, B-type natriuretic peptide; CHIP, clonal haematopoiesis of indeterminate potential; CPET, cardiopulmonary exercise testing; CTEPD, chronic thromboembolic pulmonary disease; CTEPH, chronic thromboembolic pulmonary hypertension; CTPA, computed tomography pulmonary angiography; NET, neutrophil extracellular trap(s); PE, pulmonary embolism; VAF, variant allele frequency; V/Q, ventilation–perfusion scintigraphy. Author-synthesized framework for evidence communication; not a guideline recommendation.

## 4. Clinical Evidence: CHIP and the Risk of VTE/PE

**Figure 5:**
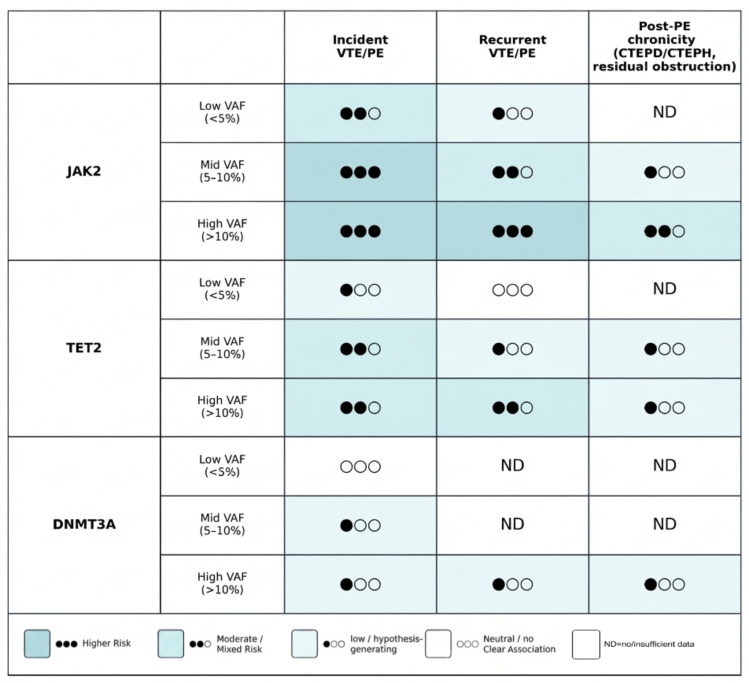
Evidence map of clinical associations between CHIP and VTE/PE outcomes by genotype, clone size, and outcome domain. Author-synthesized schematic summarizing the key clinical evidence discussed in Section 4, organized by CHIP genotype (e.g., JAK2, TET2, DNMT3A), clone size (VAF strata), and outcome domain (incident VTE/PE, recurrent VTE/PE, and post-PE chronicity endpoints). Symbols indicate the direction of association and the relative certainty/consistency of the clinical evidence (●●● higher; ●●○ moderate/mixed; ●○○ low/hypothesis-generating; ○○○ Neutral/no Clear Association); “ND” denotes no/insufficient data for a given genotype–outcome pairing. Evidence for post-PE chronicity endpoints is generally emerging and should be interpreted as largely hypothesis-generating unless supported by prospective PE-focused clinical studies. VAF strata are illustrative and may not be directly comparable across sequencing platforms and bioinformatics pipelines.

Level 1 evidence from large population cohorts with exome sequencing or targeted panels suggests that CHIP is associated with a modest increase in incident thromboembolic events, including PE. These Level 1 data predominantly derive from composite VTE endpoints; PE-specific outcomes and post-PE chronicity endpoints are comparatively underrepresented and should be interpreted as emerging rather than definitive. The key clinical evidence is summarized as an evidence map in Figure 5, organized by CHIP genotype, VAF strata, and outcome domain (incident VTE/PE, recurrence, and post-PE chronicity endpoints). In the ARIC cohort of older adults, CHIP was detected in approximately one quarter of participants (depending on sequencing depth, gene panels, and VAF thresholds) and was associated with an increased risk of VTE [15,20,21]. Meta-analytic data have suggested a pooled hazard ratio around 1.5, but estimates vary with study design and CHIP definitions, and between-study heterogeneity is substantial—a finding that is biologically plausible given the mixture of genotypes with divergent thrombotic effects [21].

JAK2-CHIP, although uncommon, carries the strongest and most reproducible thrombotic signal. Case–control and cohort analyses support an association between JAK2V617F-positive clonal hematopoiesis and incident VTE, consistent with experimental work demonstrating JAK2-driven NETosis and venous thrombosis [14,25,26].

TET2-CHIP appears to confer a moderate but recurring association with VTE. In ARIC, TET2 mutations were associated with higher VTE risk, while meta-analytic estimates have been more modest and at times non-significant [20,21,27,28,29]. These findings are consistent with a primarily inflammatory mechanism that amplifies immunothrombotic pathways rather than acting as a single dominant thrombotic trigger.

By contrast, DNMT3A-CHIP (the most prevalent driver) is frequently neutral with respect to VTE/PE risk in population analyses [20,21,27,29,30]. This underscores the danger of treating “CHIP-positive” as a homogeneous risk label in PE.

Clone size also matters. Associations between CHIP and thrombotic outcomes are generally stronger in the presence of larger clones (e.g., VAF ≥ 10%) [14,31]. In myeloproliferative contexts, higher JAK2V617F allele burden has been linked to markedly increased VTE risk, supporting the principle that biological “dose” modulates thrombotic phenotype [32].

VAF thresholds should be interpreted as study-level stratification tools rather than universal clinical cutoffs. VAF is inherently platform- and bioinformatics pipeline–dependent (sequencing depth, error suppression/UMI use, variant caller and filtering parameters), and is influenced by specimen type and clonal architecture. Consequently, the same biological clone can yield different reported VAFs across assays and laboratories, particularly at low VAF near assay-specific limits of detection/quantification. We therefore caution against using fixed VAF cut points as decision thresholds; VAF is best interpreted in context and, where clinically relevant, supported by confirmatory testing and standardized reporting.

Most epidemiologic analyses use composite VTE endpoints rather than PE-specific or post-PE chronicity outcomes, and effect estimates vary with age distribution, sequencing depth, VAF thresholds, and adjustment for comorbidity. Accordingly, in Figure 5 we separate incident events from recurrence and post-PE chronicity outcomes to make explicit where evidence is robust versus still emerging. CHIP also clusters with exposures and conditions that independently influence thrombosis (e.g., smoking, malignancy or prior chemotherapy, chronic inflammatory states), and some detected variants may represent early or evolving myeloid disease rather than isolated CHIP. Accordingly, current evidence supports CHIP as a biologically plausible modifier of thrombotic phenotype, but not yet as a stand-alone clinical risk determinant in PE. Importantly, direct human evidence linking CHIP to impaired thrombus resolution after an index PE or to progression into CTEPD/CTEPH remains limited; therefore, mechanistic arguments and CTEPH translational data should be viewed as supportive, not definitive.

Interpretation requires caution: CHIP co-segregates with age, smoking, cancer exposure, and systemic inflammation, raising the possibility that CHIP can act as both a causal driver and a biomarker of an adverse inflammatory milieu. Analyses that incorporate clone size, longitudinal VAF dynamics, mediator biomarkers (e.g., NET and fibrinolysis readouts), and robust confounder control will be critical to distinguish these roles.

Three practical interpretive principles for PE are worth emphasizing: (i) PE is not interchangeable with composite VTE endpoints; mechanisms that govern thrombus non-resolution and chronic sequelae may differ from those that trigger the index event. (ii) “CHIP-positive” is not a single biological entity; genotype and VAF determine direction and magnitude of thrombo-inflammatory effects. (iii) The clinically relevant question in PE-to-CTEPD/CTEPH is not only who forms a clot, but who fails to clear it despite anticoagulation—a setting in which immunothrombosis and NET biology are plausible modifiers.

From incidence to chronicity—a testable prediction. If CHIP primarily acts by shifting thrombus composition toward a NET-rich, dense fibrin phenotype and by sustaining low-grade vascular inflammation, its impact could be disproportionately visible in endpoints that reflect persistence (residual perfusion defects, recurrent PE on anticoagulation, CTEPD/CTEPH) rather than in incident VTE alone. This predicts stronger genotype- and VAF-dependent signals in prospective post-PE cohorts that measure thrombus resolution and NET/fibrinolysis biomarkers longitudinally.

## 5. CTEPH as a Translational Model

CTEPH provides a unique translational window into the PE-to-chronic disease continuum. Unlike acute PE, where biological characterization of the thrombus is limited, CTEPH offers time, access to disease material, and stable clinical phenotypes. Together, these features allow more direct interrogation of why thrombus resolves in some patients but becomes organized and persistent in others [33,34].

A recurring challenge is attribution: persistent or recurrent obstruction may reflect residual organized embolic material, recurrent embolization, or de novo in situ thrombosis on a remodeled pulmonary vascular bed. Most cohorts cannot fully separate these mechanisms, and definitions of CTEPD/CTEPH, imaging adjudication, and anticoagulation exposure are heterogeneous—factors that should temper causal language when linking CHIP to chronicity.

CTEPH is not simply the consequence of mechanical obstruction of proximal pulmonary arteries. It also involves small-vessel remodeling and microangiopathy in territories that may not appear obstructed, contributing to progressive pulmonary hypertension, right ventricular dysfunction, and functional decline [33,35]. The clinical reality that anticoagulation alone often fails to halt disease progression supports a pathogenetic role for inflammation, innate immunity, and maladaptive vascular remodeling [33,36,37].

Level 2 evidence from clinical CTEPH studies indicates that variants consistent with clonal hematopoiesis are present in a meaningful proportion of patients, particularly with advancing age. In one multicenter cohort, approximately 9% of patients carried a CHIP mutation (VAF ≥ 2%), most commonly DNMT3A, TET2, ASXL1, and JAK2 [16]. A separate Japanese study reported clonal hematopoiesis in roughly one fifth of patients, rising substantially in the oldest age strata [17].

Beyond prevalence, the most clinically relevant observation is phenotype severity. Clonal hematopoiesis has been associated with worse functional status, higher markers of right ventricular strain, and greater need for supportive measures such as home oxygen therapy [16,17]. Importantly, some studies report imaging findings and clinical courses suggestive of fresh thrombus layering and/or ongoing in situ pulmonary artery thrombosis despite anticoagulation, accompanied by a greater requirement for additional balloon pulmonary angioplasty sessions in CHIP-positive patients [17]. These observations should be interpreted cautiously because definitions of “re-thrombosis” vary across cohorts, but they strengthen the hypothesis that CHIP can mark a persistent immunothrombotic activity state in chronic thromboembolic disease.

Mechanistic coherence is strengthened by molecular signatures. Transcriptomic analyses have shown enrichment of coagulation and NET-related pathways in patients with clonal hematopoiesis, accompanied by higher circulating markers of NETs (e.g., citrullinated histone H3) [17]. Proteomic studies of platelets in CTEPH have similarly highlighted differential expression of proteins involved in complement/coagulation cascades and NET formation, with NET-related markers correlating with hemodynamic severity and right ventricular dysfunction [38].

## 6. Mechanistic Axes Linking CHIP to Thrombus Persistence in the PE-to-CTEPD/CTEPH Continuum

The clinical question in post-PE care is not only who forms a clot, but who fails to clear it. We propose that selected CHIP genotypes (most consistently TET2 and JAK2, and in a clone-size (VAF)-dependent manner) bias innate immune effector programs toward immunothrombosis. This can alter thrombus microstructure and the surrounding vascular milieu, reducing susceptibility to endogenous fibrinolysis and favoring organization, fibrosis, and chronic obstruction. Below we outline convergent pathways and highlight genotype-resolved mechanistic axes that are testable in prospective post-PE cohorts and in CTEPH translational studies. For a pragmatic overview of these mechanistic axes—and their expected thrombus, biomarker, and clinical correlates—we provide a summary map in Supplementary Table S2.

In simplified terms, CHIP alters the functional program of myeloid cells. This increases the likelihood of inflammatory and pro-thrombotic effector responses, including NET formation, monocyte tissue factor expression, cytokine release, and endothelial–platelet activation. These changes can couple innate immunity to coagulation and may shift thrombus microstructure toward dense fibrin networks interlaced with DNA and histones features associated with mechanical stiffness and relative resistance to fibrinolysis.

Inflammation-to-coagulation coupling provides additional molecular granularity. CHIP-skewed monocytes can increase procoagulant activity via tissue factor expression, microparticle shedding, and amplified thrombin generation. Activated neutrophils and platelets form aggregates that concentrate coagulation factors and promote endothelial activation, while NET components (DNA, histones, neutrophil proteases) provide a scaffold for platelet adhesion, support contact-pathway activation, and can sequester or inhibit fibrinolytic proteins (e.g., plasminogen/tPA). In parallel, chronic inflammation may shift the fibrinolytic balance toward persistence through upregulation of plasminogen activator inhibitor-1 (PAI-1) and formation of densely cross-linked fibrin. Together, these convergent effects can yield a mechanically reinforced, fibrinolysis-resistant thrombus phenotype that persists even when anticoagulation prevents new clot propagation.

### 6.1. The TET2 Axis: Inflammasome Activation and IL-1β/IL-6 Signaling

TET2 regulates epigenetic programs in myeloid cells. Loss of TET2 function promotes a more inflammatory macrophage/monocyte phenotype with heightened NLRP3 inflammasome activity and increased IL-1β production, with downstream engagement of IL-6 signaling and endothelial activation [12,39,40,41]. In the pulmonary vascular context, sustained cytokine exposure can amplify leukocyte recruitment, endothelial dysfunction, and tissue-factor-driven thrombin generation, tilting thrombus fate from resolution toward organization and fibrosis. This axis is mechanistically druggable (e.g., IL-1β pathway modulation, upstream inflammasome targeting), but any translation to PE/CTEPD/CTEPH remains unproven and should be tested using target-engagement biomarkers rather than inferred from incident VTE associations alone (Level 3).

### 6.2. The JAK2 Axis: A Highly Thrombogenic, MPN-like Phenotype

JAK2-associated CHIP, particularly JAK2V617F, is linked to a markedly thrombogenic, MPN-like myeloid state even in the absence of overt myeloproliferative disease. Mechanistically, JAK2 activation can prime neutrophils, platelets, and endothelium toward adhesion (integrin activation), platelet–neutrophil aggregate formation, and NET release. NETs provide a DNA-histone scaffold that binds platelets and von Willebrand factor, concentrates coagulation factors, and can amplify both thrombin generation and contact-pathway activation. NET components (histones, neutrophil proteases) densify fibrin, increase clot stiffness, and reduce permeability, creating relative resistance to tissue plasminogen activator (tPA)-mediated lysis. These features make JAK2-CHIP a biologically plausible contributor to post-PE persistence and chronic obstruction. Candidate modifiable nodes include NET formation/clearance (e.g., PAD4-DNA axis), platelet–leukocyte adhesion pathways, and contact-pathway inhibition, but clinical translation in isolated JAK2-CHIP requires trials and careful safety evaluation (Level 3).

### 6.3. Other CHIP Genotypes and Convergent Pathways

Not all CHIP genotypes appear equally thrombogenic, and several signals may reflect background inflammaging or treatment-related exposures. Isolated DNMT3A-CHIP is common and often shows weak or neutral associations with PE/VTE in population analyses, particularly at low VAF, suggesting that it may more often behave as a marker of hematopoietic aging than a dominant thrombotic driver. ASXL1 and spliceosome-gene mutations have been linked to inflammatory myeloid skewing in some settings, but PE/CTEPH-specific data remain limited. PPM1D and TP53 variants, frequently therapy-related, warrant careful clinical context because their presence can co-segregate with cancer history and cytotoxic exposures that independently influence thrombosis. Accordingly, genotype-resolved reporting (including co-mutations and clone multiplicity) is essential when interpreting CHIP in post-PE cohorts and CTEPH registries.

Taken together, genotype-specific clonal inflammation (most consistently TET2 and JAK2) can converge on a NET-centered effector program and on downstream modules that stabilize thrombus and impair clearance [42,43]. In addition to NETosis, two pathway modules are particularly relevant to thrombus persistence: (i) complement and contact/coagulation--inflammation crosstalk that amplifies tissue factor signaling, platelet–endothelial activation, and thrombin generation, and (ii) a shift in the fibrinolysis-organization balance (e.g., PAI-1/TAFI, fibrin microstructure, and fibroproliferative remodeling) [44]. These axes provide a coherent biological bridge from acute PE to thrombus non-resolution and chronic thromboembolic disease [15]. Key uncertainties remain: the relative contribution of recurrent embolization versus de novo in situ thrombosis, the timing of NET-dominant phases during clot organization, and the extent to which CHIP acts as a driver rather than a biomarker of an adverse inflammatory milieu [15]. These questions are addressable with prospective, mediator-informed studies that pair genotype and VAF with longitudinal biomarker panels and standardized post-PE imaging and clinical endpoints.

### 6.4. Complement and Coagulation–Inflammation Crosstalk: Amplifying Loops in Thrombus Persistence

Complement activation can reinforce immunothrombosis by promoting leukocyte recruitment, endothelial activation, and platelet–leukocyte interactions, thereby sustaining thrombin generation and intrathrombus inflammation during organization [45]. In parallel, contact pathway signaling (FXI/FXII) and protease-activated receptor (PAR) pathways provide additional routes by which inflammatory cues can translate into sustained coagulation and microvascular obstruction. Although direct CHIP → complement evidence in PE/CTEPH is limited, CHIP-skewed myeloid programs that increase cytokine and tissue-factor tone are biologically compatible with these amplifying loops and may help explain persistent, inflammation-rich thrombus biology in susceptible patients. Operationally, this axis can be probed with plasma complement readouts (e.g., C3a/C5a and sC5b-9), markers of thrombin generation, and contact-pathway activity (FXI/FXII), sampled serially during the early post-PE organization window (for example, the first 6–12 weeks) and related to imaging-defined residual obstruction.

### 6.5. Fibrinolysis-Organization Balance: Inhibitors, Fibrin Microstructure, and Remodeling

Failure of thrombus clearance reflects not only ongoing thrombin generation but also an unfavorable balance between endogenous fibrinolysis and the organization/fibrosis program. A NET- and histone-enriched thrombus can alter fibrin architecture and increase resistance to plasmin-mediated lysis, while fibrinolysis inhibitors (including PAI-1 and TAFI) and platelet-derived mediators may further suppress clot breakdown [46]. Over time, this shifts the lesion toward collagen-rich, fibrocellular remodeling with endothelial and smooth muscle responses that are central to CTEPD/CTEPH. Future studies should therefore pair NET markers with quantitative fibrinolysis measures and inhibitor profiling to test whether CHIP is associated with a reproducible “low-fibrinolysis/high-organization” signature after PE. Practical assays include clot-lysis time or thromboelastography/viscoelastic testing with a tPA challenge, plasmin generation, and activity/antigen profiling of PAI-1 and TAFI, ideally paired with NET markers (cfDNA and citrullinated histones) in the same time window. Sampling at baseline, 4–8 weeks, and 3–6 months after PE would allow testing whether a stable low-fibrinolysis/high-organization signature tracks with non-resolution [45].

### 6.6. Differential Mechanistic Predictions: Non-Resolution vs. Recurrent Emboli vs. In Situ Thrombosis

A key interpretive challenge in chronic thromboembolic disease is distinguishing persistent obstruction driven by thrombus non-resolution from (i) recurrent embolization and (ii) de novo in situ thrombosis on a remodeled pulmonary vascular bed. CHIP may plausibly contribute to more than one mechanism, but a genotype- and mediator-informed approach can generate differentiating, testable predictions.

If CHIP primarily amplifies non-resolution biology (a “fibrinolysis-resistant clot” phenotype), we would predict the following: (a) stronger associations with residual perfusion defects and organized thrombus burden than with first-ever VTE; (b) a mediator profile enriched for NET and fibrinolysis–inhibition signals (e.g., NET markers, PAI-1-dominant balance) that tracks with impaired resolution over time; and (c) ex vivo thrombus properties consistent with dense fibrin/DNA architecture and relative resistance to tPA-mediated lysis (Level 3).

If recurrent embolization is dominant, associations should align more closely with systemic recurrence risk and venous-source phenotypes (e.g., DVT burden, provoking factors) and may be less tightly coupled to pulmonary-local NET signatures. Conversely, if in situ thrombosis is dominant, we would predict pulmonary-vascular-local activation signatures despite therapeutic anticoagulation–platelet–neutrophil aggregates, endothelial activation, and potentially contact-pathway/coagulation-complement enrichment together with imaging patterns compatible with fresh thrombus layering (Levels 2–3).

These differentiating predictions motivate study designs that jointly capture (i) CHIP genotype/VAF, (ii) serial mediator panels (NETs, coagulation/complement activation, fibrinolysis balance, platelet–endothelial activation), and (iii) standardized adjudication of recurrence, residual obstruction, and in situ re-thrombosis using harmonized imaging definitions (Level 2).

## 7. Genotype-Informed Phenotyping: A Pragmatic Proposal

To become clinically useful in thromboembolic medicine, CHIP must be interpreted as a genotype- and clone-size-defined thrombo-inflammatory endophenotype rather than a binary label (“CHIP present/absent”). In PE and CTEPH, the most relevant outputs extend beyond the index event to recurrence, thrombus persistence/non-resolution, and progression along the PE-to-CTEPD/CTEPH continuum. Current data support a genotype-informed approach, with VAF and clinical context as essential modifiers [21,47,48]. A pragmatic, time-resolved phenotyping strategy after index PE—including CHIP genotyping, serial VAF assessment, NET/fibrinolysis biomarkers, and harmonized imaging/hemodynamic endpoints—is outlined in Figure 4. Because CHIP effects are strongly genotype- and clone-size dependent, we propose a hypothesis-generating genotype-by-VAF matrix to guide risk stratification and trial enrichment (Figure 3).

Operationalizing this framework in studies (and, cautiously, in expert-center practice) requires a minimal common dataset. To support pragmatic triage in referral practice, we outline clinical scenarios in which CHIP testing may be considered in PE/CTEPD/CTEPH, along with minimal co-testing and common interpretation pitfalls (Supplementary Table S3). Candidate populations include unprovoked or recurrent PE, PE with disproportionate residual perfusion limitation on follow-up, and CTEPD/CTEPH with suspected ongoing thrombotic activity or unexpectedly limited response after pulmonary endarterectomy (PEA) or balloon pulmonary angioplasty (BPA). CHIP testing should be reported with gene, exact VAF, and co-mutation/clone multiplicity, alongside longitudinal blood counts and evaluation for CCUS or overt myeloid neoplasia when appropriate. Endpoints should extend beyond recurrent VTE to structured post-PE follow-up (symptoms and exercise capacity at ~3–6 months), repeat imaging for residual perfusion defects, progression to CTEPD/CTEPH, functional capacity (6 min walk distance and/or cardiopulmonary exercise testing), right ventricular strain biomarkers (e.g., natriuretic peptides), and imaging markers of thrombus persistence and remodeling.

### 7.1. Phenotype A: JAK2-CHIP (High Thrombogenicity)

When to consider: recurrent or unprovoked PE/VTE disproportionate to recognized risk factors, thrombosis despite apparently adequate anticoagulation, or hematologic “red flags” (borderline thrombocytosis, leukocytosis, or rising hematocrit) [8,14,49,50].

Biological interpretation: among CHIP genotypes, JAK2 carries the strongest thrombotic signal and aligns with a highly thrombogenic, MPN-like biology that includes integrin activation and NETosis [8,14,51].

Practical considerations (research/expert-center): a JAK2-CHIP result should prompt hematology collaboration to evaluate for overt myeloproliferative neoplasms when clinical or laboratory features raise suspicion. Risk interpretation should remain contextual and incorporate VAF, blood counts over time, provoking factors, and competing drivers of thrombosis; outside trials, management should follow established PE/VTE standards.

### 7.2. Phenotype B: TET2-CHIP (Inflammatory Modifier of Recurrence and Chronicity)

When to consider: older patients with unprovoked or weakly provoked PE, persistent post-PE symptoms, or chronic thromboembolic disease with signs of biological activity (elevated inflammatory markers, suspected re-thrombosis, or suboptimal response after PEA/BPA) [15,16,20].

Biological interpretation: TET2-CHIP aligns with inflammasome-driven inflammation (IL-1β/IL-6), endothelial activation, and immunothrombosis [12,14,25,39,52,53,54]. In CTEPH cohorts, clonal hematopoiesis has been associated with higher NET markers and signals consistent with ongoing thrombotic activity despite anticoagulation [15,39,42].

Practical considerations (research/expert-center): treat TET2-CHIP as hypothesis generating. It may justify closer structured follow-up and inclusion in biomarker-driven registries (NET markers, fibrinolysis readouts, and post-PE chronicity endpoints), but it should not be used as a stand-alone trigger to change anticoagulation strategy or other standard management.

### 7.3. Phenotype C: DNMT3A-CHIP (Common, Often Neutral for PE)

DNMT3A-CHIP is the most prevalent CHIP genotype and, in most population analyses, shows weak or neutral associations with VTE/PE compared with TET2 or JAK2 [5,20,21]. In PE/CTEPH, isolated DNMT3A at low VAF is best interpreted as a non-specific marker of hematopoietic aging rather than a high-risk thrombotic phenotype [5,20,21,55,56].

Clinical implication: DNMT3A-CHIP does not, by itself, justify deviation from standard PE/CTEPH management or intensified hematologic surveillance unless accompanied by cytopenias, co-mutations, cancer-related exposure, or other clinical concerns.

In sum, genotype-informed phenotyping provides a practical framework: JAK2-CHIP flags a highly thrombogenic phenotype; TET2-CHIP suggests an inflammatory, potentially chronicity-prone phenotype; and DNMT3A-CHIP is commonly neutral in PE. Prospective models should replace “CHIP yes/no” with genotype- and VAF-aware variables [56].

## 8. Diagnostic Implications: Should We Test for CHIP in PE/CTEPH?

At present, there are no guideline recommendations for routine CHIP screening in patients with PE, CTEPD, or CTEPH. The evidence base is biologically compelling but not yet sufficiently prospective or PE-specific to justify routine testing; targeted testing is best restricted to research protocols and expert-center registries [15,17,57,58].

### Limitations of the Current Evidence and of This Review

The literature supports biological plausibility and consistent signals for selected genotypes (notably JAK2 and TET2), but several limitations constrain clinical inferences in PE/CTEPD/CTEPH at present.

First, much of the epidemiologic evidence links CHIP to composite VTE rather than PE-specific endpoints, and relatively few studies adjudicate PE recurrence, clot burden, right ventricular dysfunction, or PE-to-CTEPD/CTEPH progression as primary outcomes. Extrapolation from VTE to PE phenotypes should therefore remain cautious.

Second, CHIP is not a binary exposure: associations vary by genotype, clone size (VAF), and clonal architecture (multiple clones/co-mutations). Technical heterogeneity (gene panels, sequencing depth, error-correction, VAF thresholds) limits cross-study comparability and precludes universally valid genotype- or VAF-based clinical cutoffs.

Third, residual confounding and causality remain central concerns. CHIP co-segregates with age, cancer and treatment exposures, smoking, systemic inflammation, and cardiometabolic disease. CHIP may act as both a causal driver and a biomarker of an adverse inflammatory milieu (inflammaging); resolving this will require longitudinal VAF dynamics, mediator biomarkers (NET and fibrinolysis readouts), and formal mediation/causal inference approaches.

Fourth, outcome and phenotype heterogeneity matters. Definitions of recurrence, anticoagulation exposure, and event ascertainment vary across cohorts; in CTEPD/CTEPH, terms such as “ongoing thrombosis” or “re-thrombosis” can reflect distinct processes (fresh emboli, in situ thrombosis, or superimposed thrombus on chronic material) that are not uniformly operationalized.

Fifth, human mechanistic data remain incomplete. NET markers, cfDNA/citrullinated histones, tissue availability, and ex vivo assays are variably reported, and causal links from specific genotypes to thrombus persistence or pulmonary vascular remodeling are not yet proven in humans.

Finally, this review is narrative rather than a formal systematic review with meta-analysis. Although we used a structured search and triangulated evidence across epidemiology, mechanism, and translational CTEPH data, publication bias and selective reporting may persist; the current literature is also too heterogeneous for robust pooled estimates.

Together, these limitations support a conservative stance: CHIP testing in PE/CTEPD/CTEPH should be considered investigational and embedded in prospective studies or expert-center registries until genotype- and VAF-informed risk models and actionable interventions are validated.

Where targeted testing may be most informative (primarily in research or expert centers) includes the following: (A) CTEPH/CTEPD with suspected ongoing thrombotic activity despite anticoagulation or unexpectedly poor response after PEA/BPA; (B) older patients with unprovoked or recurrent PE, where biological risk phenotyping and structured follow-up are priorities; (C) phenotypes suggestive of JAK2-driven thrombogenicity, where results can accelerate hematology evaluation and trial eligibility [14,15,17,25,59]. For pragmatic interpretation in referral practice and research settings, we summarize the minimum elements that should be reported when CHIP is evaluated in CTEPH (Supplementary Table S4).

Minimum reporting standards for studies and registries should include the following: sequencing method and gene panel; VAF threshold and exact VAF; key genotype categories (at least DNMT3A, TET2, ASXL1, JAK2); co-mutations/clone multiplicity when available; and clinical data sufficient to exclude CCUS or overt myeloid neoplasia when appropriate [12,15,17,40,60].

Counseling and incidental findings deserve explicit attention. CHIP may carry implications beyond thrombosis, including risk of progression to hematologic neoplasia and associations with atherosclerotic and inflammatory disease. When CHIP is identified in PE/CTEPH, results should be communicated in a measured manner, interpreted in the context of blood counts and clinical features, and used to trigger appropriate hematology collaboration rather than to drive unproven treatment escalation.

## 9. Therapeutic Implications: What Changes Today, and What May Change Tomorrow

Table 2 summarizes what should not change in current PE/CTEPH management based on CHIP status, and delineates research directions that may become actionable with prospective validation.

### 9.1. Today: Standard Care Remains Standard Care

CHIP status is not currently an indication to modify standard anticoagulation strategies, including drug choice or duration. Decisions should continue to be guided by established recurrence and bleeding risk assessment, provocation status, cancer, and patient preferences [15,20,61].

The principal near-term risk is over-interpretation, particularly for isolated DNMT3A-CHIP, which may prompt unnecessary extension or intensification of anticoagulation and avoidable bleeding exposure [14,20]. In CTEPH, CHIP is not part of current selection criteria for PEA/BPA or pulmonary hypertension therapies [15].

### 9.2. Tomorrow: Anti-Inflammatory and Anti-NET Strategies

If CHIP ultimately changes practice in PE/CTEPH, it is more likely to do so through adjunctive strategies targeting thrombo-inflammation than through intensified anticoagulation [12,25,62].

The IL-1β/NLRP3 axis is a plausible target for TET2-driven clonal inflammation. Exploratory cardiovascular data suggest genotype-specific benefit of IL-1β blockade among individuals with TET2-CHIP, and colchicine has been reported to modulate clonal dynamics in secondary analyses [12,25,63,64]. However, no randomized data support these approaches in PE/CTEPH, and safety in combination with anticoagulation must be established, with particular attention to infection risk in patients with cardiopulmonary limitation [15,65].

NET-directed strategies are attractive in CTEPH given translational signals linking NET markers to disease severity and thrombus persistence. Preclinical data support DNase I (NET dismantling) and PAD4 inhibition (NET formation blockade) as proof-of-concept approaches to reduce NET-mediated thrombus stabilization, fibroinflammatory organization, and microvascular remodeling [59,62,66,67]. These remain investigational; prerequisites include standardized NET assays (pre-analytical handling, assay harmonization, and clinically meaningful thresholds), definition of the therapeutic window (acute PE vs. early chronicity), and careful evaluation of infection and bleeding risks particularly in patients receiving anticoagulation and with cardiopulmonary limitation [59,67].

Beyond IL-1β and NETs, several thrombo-inflammatory nodes are conceptually relevant to a CHIP-skewed immunothrombotic phenotype: platelet–endothelial adhesion pathways (e.g., P-selectin/vWF-mediated interactions), monocyte tissue factor and microparticle biology, contact-pathway inhibition (FXI/FXII as a potential strategy to reduce thrombosis with less bleeding), and the fibrinolysis–inhibition axis (PAI-1-dominant states). These targets are best approached through biomarker-enriched early-phase trials focused on target engagement and imaging-based persistence endpoints rather than empiric treatment escalation (Level 3).

For JAK2-CHIP, the immediate therapeutic relevance is clinical recognition and hematology partnership. Although JAK2 inhibitors reduce NET formation in experimental models and are established in myeloproliferative neoplasms, their role in isolated JAK2-CHIP without overt disease remains unproven and cannot be recommended outside trials [8,25,68].

## 10. Research Priorities

A clinically useful translation of CHIP into PE/CTEPH care will require prospective, genotype-aware programs that connect clonal metrics (gene, VAF, clone multiplicity, and longitudinal VAF dynamics) to mechanistic biomarkers (NET burden/turnover, platelet–leukocyte activation, and fibrinolysis balance) and to standardized clinical endpoints (recurrence, residual obstruction, incident CTEPD/CTEPH, functional capacity, right ventricular biomarkers, and hemodynamics when available).

Priority directions include:

- Prospective post-PE cohorts with harmonized endpoints and serial sampling to capture VAF dynamics, mediator biomarkers (NET and fibrinolysis balance), and imaging-defined thrombus persistence (Level 2).
- Paired blood-and-tissue studies in CTEPH integrating CHIP genotyping with pulmonary endarterectomy material and/or procedural phenotyping around BPA to test a clone-to-signature-to-thrombus-to-phenotype chain, including transcriptomic/proteomic NET and coagulation-complement signatures (Levels 2–3).
- Biomarker-driven interventional trials restricted to immunothrombosis-dominant phenotypes (e.g., high NET signature and/or higher-VAF TET2- or JAK2-CHIP with persistent obstruction despite anticoagulation), with early-phase endpoints focused on target engagement, imaging-based persistence measures, and careful bleeding/infection surveillance (Level 3).

## 11. Conclusions

Across available data, CHIP is best viewed as a measurable thrombo-inflammatory endophenotype whose clinical relevance in PE depends on genotype and clone size (VAF). Level 1 population cohorts provide the strongest evidence that CHIP increases incident VTE/PE risk when genotype and VAF are resolved, with the most consistent signal for JAK2 and a smaller, context-dependent signal for TET2, whereas isolated low-VAF DNMT3A-CHIP is common and often neutral for PE-focused outcomes.

For CTEPD/CTEPH, Level 2 clinical cohorts and Level 3 mechanistic evidence support a coherent, testable hypothesis: CHIP-skewed myeloid programs amplify inflammasome signaling and NET-driven, fibrinolysis-resistant thrombus architecture, contributing to thrombus persistence after PE. However, current human studies cannot yet disentangle causal direction or dominant mechanism (true non-resolution versus recurrent emboli or in situ thrombosis), underscoring the need for designs that adjudicate these pathways explicitly.

Clinically, CHIP testing in PE/CTEPD/CTEPH remains investigational and should not alter guideline-based anticoagulation or established CTEPH interventions outside research protocols. The practical near-term value is risk stratification and trial enrichment: prioritizing genotype- and VAF-aware prospective post-PE cohorts with serial mediator panels (NETs, coagulation/complement activation, and fibrinolysis balance) linked to harmonized imaging and clinical endpoints, complemented by paired blood-and-thrombus studies in CTEPH. This framework shifts CHIP from a nonspecific marker of aging to a biologically grounded, genotype-informed lens for precision studies of post-PE thrombus persistence and chronic thromboembolic disease.

## Figures and Tables

**Table 2:** What we do today vs. what may change tomorrow—pragmatic framework for PE/CTEPH management with consideration of CHIP.

Decision Domain	What We Do Today (Standard of Care)	Does CHIP Change Practice Today?	What Might Change Tomorrow (Research Hypotheses)	Prerequisite for Clinical Implementation
Anticoagulation after PE	Choice of agent and duration guided by recurrence risk and bleeding risk	No (no guideline-based recommendations)	Incorporating CHIP into recurrence-risk prediction models (genotype + VAF)	Prospective validation and development of predictive models
CTEPH treatment (PEA/BPA/medical therapy)	Selection based on anatomy, haemodynamics, and multidisciplinary team assessment	No	CHIP/NET markers as predictors of re-thrombosis and treatment response	Replication in independent cohorts
Anti-inflammatory therapies (IL-1β/NLRP3)	No indication in PE/CTEPH	No	Add-on therapy in TET2-CHIP phenotypes with a high NET signature	Biomarker-driven RCTs and robust safety assessment
Anti-NET strategies	Not part of standard care	No	Reducing immunothrombosis and re-thrombosis in CTEPH	Biomarker standardization, RCTs, and careful evaluation of infection and bleeding risk
Hematology referral/work-up	Driven by clinical indications	Yes, particularly for JAK2	More granular stratification of “MPN-like” VTE phenotypes	Clear referral criteria and inter-center collaboration

Abbreviations: BPA, balloon pulmonary angioplasty; CHIP, clonal haematopoiesis of indeterminate potential; CTEPH, chronic thromboembolic pulmonary hypertension; IL-1β, interleukin-1 beta; JAK2, Janus kinase 2; MPN, myeloproliferative neoplasm(s); NET, neutrophil extracellular trap(s); NLRP3, NLR family pyrin domain containing 3; PE, pulmonary embolism; PEA, pulmonary endarterectomy; RCT, randomized controlled trial; TET2, tet methylcytosine dioxygenase 2; VAF, variant allele frequency; VTE, venous thromboembolism.

## Data Availability

No new data were created or analyzed in this study. Data sharing is not applicable to this article.

## Supplementary Materials

The following supporting information can be downloaded at: https://www.mdpi.com/article/10.3390/ijms27062750/s1.

## Abbreviations

The following abbreviations are used in this manuscript:

ARIC	Atherosclerosis Risk in Communities study
ASXL1	additional sex combs like 1
BPA	balloon pulmonary angioplasty
CCUS	clonal cytopenia of undetermined significance
CHIP	clonal haematopoiesis of indeterminate potential
CTEPD	chronic thromboembolic pulmonary disease
CTEPH	chronic thromboembolic pulmonary hypertension
DNase I	deoxyribonuclease I
DNMT3A	DNA methyltransferase 3A
DVT	deep vein thrombosis
FXI	factor XI
FXII	factor XII
IL-1β	interleukin-1 beta
IL-6	interleukin-6
JAK2	Janus kinase 2
JAK2V617F	Janus kinase 2 V617F variant
MPN	myeloproliferative neoplasm
NET	neutrophil extracellular trap(s)
NLRP3	NLR family pyrin domain containing 3
PAD4	peptidylarginine deiminase 4
PAI-1	plasminogen activator inhibitor-1
PAR	protease-activated receptor
PE	pulmonary embolism
PEA	pulmonary endarterectomy
PPM1D	protein phosphatase, Mg^2+^/Mn^2+^ dependent 1D
PRISMA	Preferred Reporting Items for Systematic Reviews and Meta-Analyses
SF3B1	splicing factor 3B subunit 1
SRSF2	serine/arginine-rich splicing factor 2
TAFI	thrombin-activatable fibrinolysis inhibitor
TET2	tet methylcytosine dioxygenase 2
tPA	tissue plasminogen activator
TP53	tumor protein p53
U2AF1	U2 small nuclear RNA auxiliary factor 1
VAF	variant allele frequency
vWF	von Willebrand factor
VTE	venous thromboembolism
