# Supplementary material for: Clonal Hematopoiesis (CHIP) in Pulmonary Embolism and CTEPH: Evidence, Mechanisms, and Risk Stratification
Source: Int J Mol Sci. 2026 Mar 18;27(6):2750. doi: 10.3390/ijms27062750 (PMC13026496; doi:10.3390/ijms27062750)
Supplement: Supplementary file 1 [file ijms-27-02750-s001.zip › ijms-4155873-supplementary.pdf]

Clonal Hematopoiesis (CHIP) in Pulmonary Embolism and CTEPH: Evidence, Mechanisms, and Risk Stratification

Supplementary File

Content:

Table S1: Literature Search Strategy 2
Table S2: Practical map of mechanistic CHIP axes in PE/CTEPH 4
Table S3: When to consider CHIP testing in PE/CTEPD/CTEPH (pragmatic framework) 5
Table S4: CTEPH and clonal haematopoiesis (CHIP): minimum reporting and interpretation checklist for referral practice and research 6

Table S1: Literature Search Strategy

MEDLINE (PubMed)
Query A (clinical association):<br>(("Clonal Hematopoiesis"[Mesh] OR "clonal hematopoiesis"[tiab] OR CHIP[tiab] OR "clonal hematopoiesis of indeterminate potential"[tiab] OR "age-related clonal hematopoiesis"[tiab])<br>OR ((DNMT3A[tiab] OR TET2[tiab] OR JAK2[tiab] OR ASXL1[tiab] OR TP53[tiab] OR PPM1D[tiab] OR SF3B1[tiab] OR SRSF2[tiab])<br>AND (somatic[tiab] OR mutation*[tiab] OR clonal[tiab] OR clone*[tiab] OR "variant allele frequency"[tiab] OR VAF[tiab])))<br>AND<br>("Venous Thromboembolism"[Mesh] OR "Pulmonary Embolism"[Mesh] OR "Thrombosis"[Mesh]<br>OR venous thromboembol*[tiab] OR VTE[tiab] OR pulmonary embol*[tiab] OR PE[tiab] OR thromb*[tiab] OR emboli*[tiab]<br>OR "Chronic Thromboembolic Pulmonary Hypertension"[Mesh] OR CTEPH[tiab] OR CTEPD[tiab] OR "chronic thromboembolic"[tiab])<br><br>OR<br><br>Query B (mechanisms / thrombus non-resolution):<br>(("Clonal Hematopoiesis"[Mesh] OR "clonal hematopoiesis"[tiab] OR CHIP[tiab] OR "clonal hematopoiesis of indeterminate potential"[tiab])<br>OR ((DNMT3A[tiab] OR TET2[tiab] OR JAK2[tiab] OR ASXL1[tiab]) AND (somatic[tiab] OR mutation*[tiab] OR clonal[tiab] OR VAF[tiab])))<br>AND<br>(thrombus[tiab] OR clot[tiab] OR "thrombus resolution"[tiab] OR "clot resolution"[tiab] OR "thrombus persistence"[tiab] OR "residual perfusion"[tiab] OR nonresolution[tiab] OR "non-resolution"[tiab] OR organization[tiab])<br>AND<br>("Neutrophil Extracellular Traps"[Mesh] OR NET*[tiab] OR "neutrophil extracellular trap*"[tiab] OR immunothromb*[tiab]<br>OR fibrinolysis[tiab] OR plasmin*[tiab] OR "tissue factor"[tiab] OR "plasminogen activator inhibitor-1"[tiab] OR PAI-1[tiab]<br>OR inflammasome[tiab] OR NLRP3[tiab] OR "interleukin-1 beta"[tiab] OR IL1B[tiab] OR IL-1b[tiab])
Embase
('clonal hematopoiesis'/exp OR 'clonal hematopoiesis':ti,ab OR chip:ti,ab OR 'clonal hematopoiesis of indeterminate potential':ti,ab<br>OR ((dnmt3a OR tet2 OR jak2 OR asxl1 OR tp53 OR ppm1d OR sf3b1 OR srsf2):ti,ab AND (somatic OR mutation* OR clonal OR clone* OR 'variant allele frequency' OR vaf):ti,ab))<br>AND<br>('venous thromboembolism'/exp OR 'pulmonary embolism'/exp OR thrombosis/exp OR 'chronic thromboembolic pulmonary hypertension'/exp<br>OR (vte OR 'venous thromboembol*' OR 'pulmonary embol*' OR pe OR cteph OR ctepd OR 'chronic thromboembolic'):ti,ab)<br>AND<br>(('neutrophil extracellular trap formation'/exp OR net*:ti,ab OR 'neutrophil extracellular trap*':ti,ab OR

immunothromb*:ti,ab)<br>OR (fibrinolysis/exp OR plasmin*:ti,ab OR 'tissue factor'/exp OR 'tissue factor':ti,ab OR 'plasminogen activator inhibitor 1'/exp OR pai-1:ti,ab)<br>OR (inflammasome/exp OR nlrp3:ti,ab OR 'interleukin 1 beta'/exp OR il1b:ti,ab OR il-1b:ti,ab)<br>OR (thrombus*:ti,ab OR clot*:ti,ab OR 'thrombus resolution':ti,ab OR non-resolution:ti,ab OR nonresolution:ti,ab OR 'residual perfusion':ti,ab))
Web of Science Core Collection
TS=(("clonal hematopoiesis" OR CHIP OR "clonal hematopoiesis of indeterminate potential" OR "age-related clonal hematopoiesis"<br>OR ((DNMT3A OR TET2 OR JAK2 OR ASXL1 OR TP53 OR PPM1D OR SF3B1 OR SRSF2) NEAR/3 (somatic OR mutation* OR clonal OR clone* OR VAF OR "variant allele frequency")))<br>AND (VTE OR "venous thromboembol*" OR "pulmonary embol*" OR PE OR thromb* OR embol* OR CTEPH OR CTEPD OR "chronic thromboembolic")<br>AND (NET* OR "neutrophil extracellular trap*" OR immunothromb* OR fibrinolysis OR plasmin* OR "tissue factor" OR PAI-1 OR inflammasome OR NLRP3 OR "IL-1" OR thrombus* OR clot* OR "thrombus resolution" OR nonresolution OR "non-resolution" OR "residual perfusion"))
Scopus
TITLE-ABS-KEY(<br>("clonal hematopoiesis" OR CHIP OR "clonal hematopoiesis of indeterminate potential" OR "age-related clonal hematopoiesis"<br>OR ((DNMT3A OR TET2 OR JAK2 OR ASXL1 OR TP53 OR PPM1D OR SF3B1 OR SRSF2) W/3 (somatic OR mutation* OR clonal OR clone* OR VAF OR "variant allele frequency")))<br>AND (VTE OR "venous thromboembol*" OR "pulmonary embol*" OR PE OR thromb* OR embol* OR CTEPH OR CTEPD OR "chronic thromboembolic")<br>AND (NET* OR "neutrophil extracellular trap*" OR immunothromb* OR fibrinolysis OR plasmin* OR "tissue factor" OR PAI-1 OR inflammasome OR NLRP3 OR "IL-1" OR thrombus* OR clot* OR "thrombus resolution" OR nonresolution OR "non-resolution" OR "residual perfusion")<br>)
Cochrane Library
Search in Cochrane Library (CENTRAL and Reviews):<br>("clonal hematopoiesis" OR CHIP OR "clonal hematopoiesis of indeterminate potential"):ti,ab,kw<br>AND (VTE OR "venous thromboembol*" OR "pulmonary embol*" OR PE OR CTEPH OR CTEPD OR "chronic thromboembolic"):ti,ab,kw
Clinical trial registries (ClinicalTrials.gov; ICTRP)
ClinicalTrials.gov / WHO ICTRP (if available):<br>("clonal hematopoiesis" OR CHIP OR DNMT3A OR TET2 OR JAK2) AND ("pulmonary embolism" OR venous thromboembolism OR CTEPH OR chronic thromboembolic)
Hand-searching and citation chasing
1) Screen reference lists of all included full-text articles and key reviews.<br>2) Forward citation tracking for seminal CHIP-VTE/PE studies and key CTEPH/NETs/coagulation papers.<br>3) Targeted author search for high-yield groups in CHIP and CTEPH.

4) Search grey literature (conference abstracts) for signals; include only if adequate methods/results are reported.

Table S2: Practical map of mechanistic CHIP axes in PE/CTEPH

Mechanistic axis	Dominant biological driver	Most likely thrombus consequences	Potential clinical phenotype in PE/CTEPH	What to measure (research)
TET2 → inflammasome/IL-1β/IL-6	chronic myeloid inflammation	greater propensity for immunothrombosis and impaired thrombus resolution	PE in older patients, with potential chronic sequelae; an “inflammatory phenotype”	genotype + VAF; CRP/IL-6; NET biomarkers; post-PE symptom trajectory
JAK2 → “MPN-like” thrombogenicity	pronounced prothrombotic tendency with neutrophil/platelet activation	greater tendency to form thrombus, with possible recurrences	recurrent PE/VTE, seemingly “disproportionate”; need for close haematology collaboration	genotype + VAF; full blood count; exclusion of overt MPN; NET biomarkers
NET as a shared effector	immunothrombosis (neutrophil–platelet–endothelium axis)	thrombus stabilisation, lysis resistance, and organisation	CTEPH with re-thrombosis and poorer response to interventions	MPO–DNA, citH3, cfDNA; analysis of material from PEA/BPA; correlation with CHIP genotype/VAF

Abbreviations: BPA, balloon pulmonary angioplasty; cfDNA, cell-free DNA; CHIP, clonal haematopoiesis of indeterminate potential; CRP, C-reactive protein; CTEPH, chronic thromboembolic pulmonary hypertension; IL, interleukin; MPN, myeloproliferative neoplasm; MPO, myeloperoxidase; NET, neutrophil extracellular trap(s); PE, pulmonary embolism; PEA, pulmonary endarterectomy; VAF, variant allele frequency; VTE, venous thromboembolism.

Table S3: When to consider CHIP testing in PE/CTEPD/CTEPH (pragmatic framework)

Clinical scenario	Why consider (rationale)	Minimum co-testing beyond CHIP	Key result(s) to look for	How to avoid misinterpretation
CTEPH/CTEPD with re-thrombosis despite anticoagulation	Highest translational yield; raises suspicion of immunothrombosis	NET biomarkers; imaging data with a standardised definition of re-thrombosis; haemodynamics	TET2 or JAK2; higher VAF	Do not confuse disease progression with non-adherence; standardise endpoint definitions
CTEPH after PEA or BPA with limited clinical improvement	Possible “difficult clot” phenotype and chronic inflammation	6MWD/BNP; repeat V/Q scintigraphy and CTPA; inflammatory and NET biomarkers	TET2; multiclonality	Do not interpret without accounting for anatomy and the technical success of the procedure
Unprovoked or recurrent PE in older adults	CHIP is more prevalent and may modify recurrence risk and/or propensity to chronicity	CRP/IL-6; structured post-PE follow-up (symptoms, V/Q)	TET2; possibly JAK2	Do not prolong anticoagulation based on CHIP alone; risk models require prospective validation
Phenotype suggestive of JAK2-driven thrombogenicity	JAK2 shows the strongest thrombogenic signal	Serial full blood count; haematology review	JAK2 (even at low VAF)	Do not omit evaluation for overt MPN and/or CCUS when clinically indicated

Abbreviations: 6MWD, six-minute walk distance; BPA, balloon pulmonary angioplasty; BNP, B-type natriuretic peptide; CCUS, clonal cytopenia of undetermined significance; CHIP, clonal haematopoiesis of indeterminate potential; CRP, C-reactive protein; CTEPD, chronic thromboembolic pulmonary disease; CTEPH, chronic thromboembolic pulmonary hypertension; CTPA, computed tomography pulmonary angiography; IL-6, interleukin-6; JAK2, Janus kinase 2; MPN, myeloproliferative neoplasm; NET, neutrophil extracellular trap(s); PE, pulmonary embolism; PEA, pulmonary endarterectomy; VAF, variant allele frequency.

Table S4: CTEPH and clonal haematopoiesis (CHIP): minimum reporting and interpretation checklist for referral practice and research

Domain	What to report (minimum)	Why it matters in CTEPH	Common pitfalls / how to avoid them
Patient characteristics	Age, sex, BMI; smoking status; comorbidities (atherosclerosis, diabetes, chronic inflammatory conditions); malignancy and treatment (chemotherapy/radiotherapy); VTE/PE history (number of events; provoked vs unprovoked)	Age and malignancy strongly influence CHIP prevalence and VTE risk; smoking is associated with certain driver mutations; these are key confounders	Failure to adjust for confounding may spuriously attribute effects to CHIP; include these variables in multivariable analyses
Clinical CTEPH phenotype	WHO functional class; 6MWD; BNP/NT-proBNP; oxygen therapy (yes/no); quality of life (if available)	Allows distinction between "stable" CTEPH and more aggressive/persistent phenotypes in which immunothrombosis may play a larger role	Reporting haemodynamics alone without functional status loses clinical meaning when interpreting "worse response"
Haemodynamics and imaging	Right heart catheterisation: mPAP, PVR, cardiac index; echocardiography (RV strain/dysfunction); V/Q scan or CTPA: extent of disease; assessment of microangiopathy (if used)	Ensures comparability across cohorts; helps determine whether CHIP associates with greater RV burden and persistence of thrombotic lesions	Mixing CTEPD without pulmonary hypertension with CTEPH; lack of standard criteria for identifying "re-thrombosis"
CTEPH treatment	Pulmonary endarterectomy (yes/no; date; outcome); balloon pulmonary angioplasty (number of sessions); medical therapy (e.g., riociguat); anticoagulation (agent, dose, adherence)	Without treatment details, "worse response" or differences in trajectory cannot be interpreted	Omitting adherence and dosing; failure to capture treatment changes over follow-up
Re-thrombosis / progression of thrombotic burden	Pre-specify definitions: new/progressive perfusion defects on V/Q or new lesions on CTPA; time from diagnosis; events occurring despite anticoagulation	A highly translational endpoint linking CHIP to persistent immunothrombosis	No definition leads to a "soft" endpoint; use blinded imaging adjudication or clear a priori criteria
CHIP/CH detection methodology	Assay (WES vs targeted panel); gene list; sequencing depth; VAF threshold; artefact filtering; co-mutations	Reviewers will ask how clones were detected; without this, results are not comparable across studies	Different panels yield different apparent CHIP prevalence; report panel content and VAF threshold explicitly
Genotype-informed CHIP interpretation	Driver gene (at minimum: DNMT3A, TET2, ASXL1, JAK2); VAF; number of clones (single vs ≥2)	Genotype determines biological meaning; JAK2 and TET2 often carry greater thrombo-inflammatory relevance	Treating CHIP as a binary ("CHIP+") variable; omitting VAF prevents assessment of clonal burden
Excluding alternative haematological diagnoses	Full blood count (Hb/WBC/platelets); cytopenias; suspicion of MPN/MDS; haematology review when indicated	CCUS/MPN materially change risk profile and management	Misclassifying CCUS/MPN as CHIP biases results and clinical interpretation
Inflammatory background biomarkers	CRP (optional: IL-6), fibrinogen	Helps determine whether CHIP effects are independent of non-specific systemic inflammation	Non-standardised sampling timing (acute decompensation vs clinical stability)
NET biomarkers (immunothrombosis signature)	At least 1–2 markers (e.g., citH3, MPO–DNA, cfDNA); sampling time (pre/post intervention); pre-analytical handling	NETs are a mechanistic bridge between CHIP, re-thrombosis, and persistence of CTEPH lesions	NET assays are sensitive to pre-analytics; describe procedures and document intercurrent infections at sampling

Vascular/thrombus material (if PEA performed)	Histopathology description; immunohistochemistry/NET markers in tissue; correlation with CHIP (genotype + VAF)	A translational 'gold standard' linking genotype to thrombus biology in the pulmonary arteries	Lack of standardised tissue assessment; no blinded analysis; small subgroup sizes
Follow-up endpoints	Re-thrombosis; need for additional BPA; hospitalisations; change in 6MWD/BNP; mortality; bleeding complications	Provides clinical relevance and enables benefit–risk assessment	Focusing on a single endpoint; missing bleeding outcomes (critical when discussing potential 'intensification')

Abbreviations: 6MWD, six-minute walk distance; ASXL1, additional sex combs like 1; BMI, body mass index; BNP, B-type natriuretic peptide; BPA, balloon pulmonary angioplasty; CCUS, clonal cytopenia of undetermined significance; cfDNA, cell-free DNA; CH, clonal haematopoiesis; CHIP, clonal haematopoiesis of indeterminate potential; CI, cardiac index; citH3, citrullinated histone H3; CRP, C-reactive protein; CTEPD, chronic thromboembolic pulmonary disease; CTEPH, chronic thromboembolic pulmonary hypertension; CTPA, computed tomography pulmonary angiography; DNMT3A, DNA methyltransferase 3A; Hb, haemoglobin; IL-6, interleukin-6; JAK2, Janus kinase 2; MDS, myelodysplastic syndrome(s); mPAP, mean pulmonary arterial pressure; MPN, myeloproliferative neoplasm(s); MPO, myeloperoxidase; NET, neutrophil extracellular trap(s); NT-proBNP, N-terminal pro–B-type natriuretic peptide; PE, pulmonary embolism; PEA, pulmonary endarterectomy; PLT, platelet count; PVR, pulmonary vascular resistance; RHC, right heart catheterisation; RV, right ventricle / right ventricular; V/Q, ventilation–perfusion scintigraphy; VAF, variant allele frequency; VTE, venous thromboembolism; WBC, white blood cell count; WES, whole-exome sequencing; WHO FC, World Health Organization functional class.
